# AADAC protects colorectal cancer liver colonization from ferroptosis through SLC7A11-dependent inhibition of lipid peroxidation

**DOI:** 10.1186/s13046-022-02493-0

**Published:** 2022-09-26

**Authors:** Rongquan Sun, Zhifei Lin, Xiangyu Wang, Lu Liu, Meisi Huo, Rui Zhang, Jing Lin, Chao Xiao, Yitong Li, Wenwei Zhu, Lu Lu, Jubo Zhang, Jinhong Chen

**Affiliations:** 1 Department of General Surgery, Huashan Hospital & Cancer Metastasis Institute, Fudan University, No.12, Middle Urumqi Road, Shanghai, 200040 China; 2 Department of Infectious Diseases, Huashan Hospital, Fudan University, Shanghai, 200040 China

**Keywords:** Colorectal cancer, Liver colonization, AADAC, Ferroptosis, SLC7A11

## Abstract

**Background:**

Oxidative stress is a highly active metabolic process in the liver, that poses great threats to disseminated tumor cells during their colonization. Here, we aimed to investigate how colorectal cancer (CRC) cells overcome lipid peroxidation to sustain their metastatic colonization in the liver.

**Methods:**

Orthotopic colorectal liver metastasis (CRLM) and CRC liver colonization mouse models were constructed to determine the roles of lipid peroxidation and AADAC in CRC liver colonization. The levels of lipid peroxidation were detected in cells or tissues. AADAC overexpression in LMs and its clinical relevance were analyzed. The oncogenic role of AADAC in CRC liver colonization was evaluated in cell experiments.

**Results:**

Compared with primary tumors (PTs), liver metastases (LMs) showed significantly lower glutathione to oxidized glutathione (GSH/GSSG) ratio and higher malondialdehyde (MDA) levels in CRLM patients and orthotopic mouse models. Inhibition of lipid peroxidation by liproxstatin-1 promoted CRC liver colonization in mouse models. RNA-seq results revealed AADAC as the most significantly upregulated lipid metabolism related gene in LMs compared with PTs. Analyses of datasets and patient and mouse model samples confirmed that AADAC was upregulated in LMs compared with PTs, and was correlated with poor prognosis. AADAC promoted cell proliferation, and facilitated liver colonization in a mouse model by reducing ROS accumulation, which led to lipid peroxidation and ferroptosis. Mechanistically, AADAC upregulated SLC7A11 by activating NRF2 to inhibit lipid peroxidation, thereby protecting metastatic cells from ferroptosis.

**Conclusions:**

AADAC protects metastatic CRC cells from ferroptosis by inhibiting lipid peroxidation in an SLC7A11-dependent manner, thus effectively promoting their metastatic colonization and growth in the liver. Together, our findings suggest that AADAC can act as a prognostic indicator and potential therapeutic target for CRLM.

**Supplementary Information:**

The online version contains supplementary material available at 10.1186/s13046-022-02493-0.

## Background

Colorectal cancer (CRC) is the third most common malignancy and has become the second leading cause of cancer-related death globally [1]. The liver is the most favored organ for CRC metastasis, and CRC is also the most common primary cancer that metastasizes to the liver [2, 3]. Approximately 25% of CRC patients have colorectal liver metastasis (CRLM) when diagnosed, and more than 50% of CRC patients eventually develop liver metastasis [4]. Furthermore, liver metastasis remains the major cause of CRC-related death [3, 5]. A deeper insight into the precise mechanism underlying the process of CRC liver metastasis is urgently needed.

The association of cancer cells with the metastatic target organ as postulated by the ‘seed and soil’ hypothesis implies that a combination of complex biological systems facilitates the development of metastasis to the liver [3, 6]. The reciprocal interactions between tumor cells and the tumor microenvironment play important roles in engraftment, survival and tumor progression [7]. Colonization occurs as the final stage of the metastasis cascade during which disseminated cancer cells reseed and thrive at distant organs [8, 9]. In response to the challenge of a different metabolic microenvironment, cancer cells need to reprogram their metabolic phenotypes to meet nutrient requirements and gain survival competence [10, 11].

As the most common metastatic site for CRC, the liver is a metabolically active organ that plays a central role in lipid metabolism [12, 13]. However, a hyperactive lipid metabolism process in the liver can also create a metabolic microenvironment that imposes damaging oxidative stress in the states of liver diseases, as the redox state is involved in multiple courses, such as metabolic and inflammatory liver diseases, and so on [14]. Among the factors that cause oxidative stress in cells, oxidative modification of lipids in membrane bilayers, in particular lipid peroxidation, has emerged as an important regulator of cell fate, with extensive lipid peroxidation committing cells to death via a distinct cell death paradigm termed ferroptosis [15, 16]. Ferroptosis is a new form of regulated cell death characterized by the iron-dependent accumulation of lipid peroxides to lethal levels. The sensitivity of cells to ferroptosis is tightly linked to numerous biological processes, including amino acid, iron, and polyunsaturated fatty acid (PUFA) metabolism, and the biosynthesis of glutathione, phospholipids, and NADPH [17, 18]. In response to lipid peroxidation, tumor cells also use various strategies to escape from this metabolic stress and ferroptosis to sustain their survival and metastasis [19–21]. Notably, how CRC cells overcome lipid peroxidation and escape from ferroptosis to sustain their metastatic colonization in the liver has not been fully elucidated.

In this study, we found increased levels of lipid peroxidation in CRC liver metastases compared with primary tumors in both patient samples and mouse models. Further investigation revealed that arylacetamide deacetylase (AADAC), a lipid enzyme that is involved in triglyceride metabolism, played a critical role in protecting CRC cells from lipid peroxidation-induced ferroptosis. This work provides a novel therapeutic target for ferroptosis-resistance during CRC liver colonization.

## Methods

### Patients and sample collection

Fresh-frozen tissue samples including primary tumors (PTs) and liver metastases (LMs) were collected in patients with synchronous CRLM (*n* = 19) undergoing simultaneous resection at our center. A tissue microarray (TMA) including 157 pathologically confirmed CRLM patients who received staged or simultaneous resection of both PTs and LMs at our center between 2014 and 2020 was retrospectively constructed. Overall survival (OS) was defined as the time interval from hepatectomy to death or last follow-up. Recurrence free survival (RFS) was defined as the time interval from hepatectomy to first recurrence or last follow-up. R packages: dplyr, survival, and survminer were used for survival analysis and visualization. All clinical samples were collected from patients after obtaining informed consent in accordance with a protocol approved by the Ethics Committee of Huashan hospital, Fudan University (Shanghai, China).

### Cell lines

Human CRC cell lines (HCT116, HT29, SW480, and CACO2), human embryonic kidney 293 T cells, were obtained from Cell Bank of Chinese Academy of Sciences (Shanghai, China). All the cell lines were maintained in DMEM (Gibco) supplemented with 10% (v/v) FBS at 37 °C in 5% CO_2_. For cell proliferation assay, DMEM supplemented with 5% (v/v) FBS was used.

### Mice models

All mice in use were 5 weeks old male nude mice (BALB/c nu/nu), obtained from Laboratory Animal Department of Fudan University (Shanghai, China), and were maintained on a 12 h light dark cycle at 22 °C. Animal care procedures and methods (approved by the institutional review board of Department of Laboratory Animal Science, Fudan University) were strictly abided by.

For orthotopic CRC model with spontaneous liver metastasis, CRC cells (CACO2) were cultured to reach appropriate density before use. Cells were then subcutaneously injected into nude mice. Subcutaneous xenograft tumors derived from above cells were then dissected into 1mm^3^ sections and cecally planted into nude mice. For CRC liver colonization model, CRC cells (SW480 control, SW480 ADOE, SW480 ADSL, HCT116 shNC, HCT116 sh-AADAC) (1 × 10^6^/100 μL PBS) were directly injected into spleens of nude mice, as previously described [22].

### Glutathione to oxidized glutathione ratio (GSH/GSSG Ratio) measurement

Cellular and tissue sample GSH/GSSG ratio was determined using the GSH/GSSG Ratio Detection Assay Kit (ab138881, Abcam), according to manufacturer’s instructions.

### Malondialdehyde (MDA) measurement

Cellular and tissue sample MDA concentration was determined using the Lipid Peroxidation MDA Assay Kit (S0131S, Beyotime), according to manufacturer’s instructions.

### RNA sequencing (RNA-seq) and GO analysis

RNAs were extracted from paired PTs and LMs from 3 CRLM patients using Trizol reagent, then were quantified and qualified. Sequencing libraries were generated using NEBNext® UltraTM RNA Library Prep Kit for Illumina® (NEB, USA) manufacturer’s recommendations. PCR products were purified (AMPure XP system) and library quality was assessed on Agilent Bioanalyzer 2100 system. TruSeq PE Cluster Kit v3-cBot-HS (Illumina) was used for later cluster generation. The library preparations were then sequenced on Illumina Novaseq6000 platform to generate 150 bp paired-end reads raw data (raw reads). Quality control were performed on all the raw data before downstream analyses. Star was used to align reads to reference genome [23]. HTSeq v0.6.0 was used to count the reads number mapped to each gene. DEseq2 was applied to filter the differentially expressed genes (criteria: |log2FC|> 1 and *p* value < 0.005) [24]. ClusterProfiler was used for GO analysis [25].

### Quantitative reverse transcription-polymerase chain reaction (qRT-PCR)

TRIzol reagent (15,596–018, Life Technologies) was used for total RNA extracting, cultured cells and tissue samples were washed by 1 × PBS before use. 5 × PrimeScript Buffer (RR036A-1, TAKARA) was used for cDNA reverse-transcribing. Gene expression was analyzed by SYBR Green RT-qPCR system (RR420, TAKARA) on the QuantStudio 6 Flex Real-Time PCR Platform. Relative expression levels of genes of interest were calculated according to the 2^−ΔΔCt^ method. GAPDH was taken as internal control for normalization. Primers used for qRT-PCR were listed in Supplementary Table 5.

### Immunoblotting

For immunoblotting, cell monolayers and tissues were lysed in a protease inhibitor cocktail supplemented RIPA buffer, protein concentration of cell lysates were then determined by a bicinchoninic acid (BCA) protein assay. Samples were denatured at 95 °C for 10 min with SDS-PAGE protein loading buffer before resolving by 8–12% Bis–Tris protein gels, then transferred to NC membrane. NC membrane with protein were blocked using 5% non-fat milk in TBST. Incubation of primary antibodies were done at 4 °C overnight, and secondary antibodies at room temperature for 1 h. Amersham Imager600 was use for signal detection. Information of antibodies used in this research are shown in Supplementary Table 6.

### Immunohistochemistry (IHC)

Fresh tissues were collected, preserved in 4% formaldehyde, and embedded in paraffin. Tissues were then mounted onto poly-L-lysine coated slides, dewaxed and immersed in endogenous peroxidase blocker. Microwave method was used for later antigen retrieval. Graded concentrations of antibodies were tested in preliminary experiments to determine optimal sensitivity and specificity. IHC staining was assessed by two independent pathologists with no prior knowledge of the patient characteristics. Discrepancies were resolved by consensus. The staining extent score was on a scale of 1 (grade 1) to 4 (grade 4), representing the staining intensity of weak, modest, high, and strong positive (weak and modest were classified as low expression level, while high and strong positive were classified as high expression level), corresponding to the percentage of immunoreactive tumor cells and the staining intensity. IHC H-score was computed as the sum of 4 × (% 4) + 3 × (% 3) + 2 × (% 2) + 1 × (% 1).

### Cell proliferation and viability assays

For cell proliferation assay, cells were seeded on a 96-well plate at a density of 1 × 10^3^ cells per well, and CCK-8 assay was performed at 5 time points (0, 24, 48, 72, 96 h) after seeding to evaluate cell proliferation. Cells were incubated in 5% CCK-8 (B34304, Bimake) diluted by DMEM without FBS for 1.5 h before each time point, and the absorbance at 450 nm wavelength was tested to evaluate cell proliferation of each well.

For 5-Ethynyl-2'-deoxyuridine (EdU) staining, cells were seeded on rounded glass cell slides in a 24-well plate for 24 h, then cells were stained using Yefluor 594 Edu imaging Kits (40276ES60, YEASEN) following the manufacturer’s instruction.

For cell viability assay, cells were seeded on a 96-well plate at a density of 1 × 10^4^ cells per well for 24 h, then were treated with or without graded concentrations of erastin for 24 h. Subsequently, cells were incubated in 5% CCK-8 diluted by DMEM without FBS for 1.5 h, and the absorbance at 450 nm wavelength was tested to evaluate cell viability of each well.

### Colony formation assay

Cells were seeded on a 12-well plate at a density of 1000 cells per well and cultured for 14 days with DMEM (10% FBS). At 14^th^ day, cells were washed by 1 × PBS and fixed by 4% paraformaldehyde for 30 min, then were stained by 0.1% crystal violet (W:V) for 20 min, and relative cells colonies were calculated.

### LC–MS/MS analysis

LC–MS/MS analyses were performed using an UHPLC system (1290, Agilent Technologies), equipped with a Kinetex C18 column (2.1 * 100 mm, 1.7 μm, Phenomen). The mobile phase A consisted of 40% water, 60% acetonitrile, and 10 mmol/L ammonium formate. The mobile phase B consisted of 10% acetonitrile and 90% isopropanol, which was added with 50 mL 10 mmol/L ammonium formate for every 1000 mL mixed solvent. The analysis was carried with elution gradient as follows: 0 ~ 12.0 min, 40% ~ 100% B; 12.0 ~ 13.5 min, 100% B; 13.5 ~ 13.7 min, 100% ~ 40% B; 13.7 ~ 18.0 min, 40% B. The column temperature was 55 °C. The auto-sampler temperature was 4 °C, and the injection volume was 2 μL.

The QE mass spectrometer was used for its ability to acquire MS/MS spectra on data-dependent acquisition (DDA) mode in the control of the acquisition software (Xcalibur 4.0.27, Thermo). In this mode, the acquisition software continuously evaluates the full scan MS spectrum. The ESI source conditions were set as following: sheath gas flow rate as 30 Arb, Aux gas flow rate as 10 Arb, capillary temperature 320 °C(positive), 300 °C (negative), full MS resolution as 70,000, MS/MS resolution as 17,500, collision energy as 15/30/45 in NCE mode, spray Voltage as 5 kV (positive) or -4.5 kV (negative), respectively.

The raw data files were converted to files in mzXML format using the ‘msconvert’ program from ProteoWizard. Peak detection was first applied to the MS1 data. The CentWave algorithm in XCMS was used for peak detection with the MS/MS spectrum, lipid identification was achieved through a spectral match using LipidBlast library.

R package pheatmap was used for lipid species visualization.

### Lipid reactive oxygen species (ROS) measurement

For Dichloro-dihydro-fluorescein diacetate (DCFH-DA) staining, cells were seeded in 6 wells plates for 24 h, then were incubated with DCFH-DA using ROS Assay Kit (S0033S, Beyotime) following the manufacturer’s protocol. After staining, Relative intensity of ROS was evaluated by flow cytometry promptly.

For boron-dipyrromethene (BODIPY) staining, cells were seeded in 6 wells plates 24 h before administration of indicated agents. The cells were then incubated with 10 μM TBHQ (S4990, Selleck) for 1 h or with 5 μM ML385 (S4990, Selleck) for 48 h, then treated cells with 15 μM erastin (S7242, Selleck). Cells were subsequently washed, lysed and collected, stained with 5 μM BODIPY 581/591 (D3861, Thermo Fisher Scientific) for 30 min at 37 °C. Cells were then washed and resuspended with PBS, and analyzed by flow cytometry promptly. The intensity shift of fluorescence emission from 590 nm (non-oxidized channel) to 510 nm (oxidized channel) were analyzed.

### Oil red O (ORO) staining

Cells were seeded on round coverslips in 12 well plate overnight, then washed 3 times with PBS and fixed with 4% (w:v) paraformaldehyde for 25 min. Cells were then stained with 3 mg/mL Oil Red O (O0625, Merck) solution in 60% isopropanol (v:v) for 30 min, and counterstained with hematoxylin (C0107, Beyotime).

### Statistical analysis

Analyses of experimental data were detailed in figure legends. GraphPad Prism 9.0.2 was used for evaluation of statistical significance. SPSS Statistics 20 was used for patient clinical information analysis. R version: 4.0.5. Rstudio version: 1.3.1093. *p* < 0.05 was considered as statistically significant.

## Results

### Lipid peroxidation restrains CRC liver colonization

**Fig. 1:**
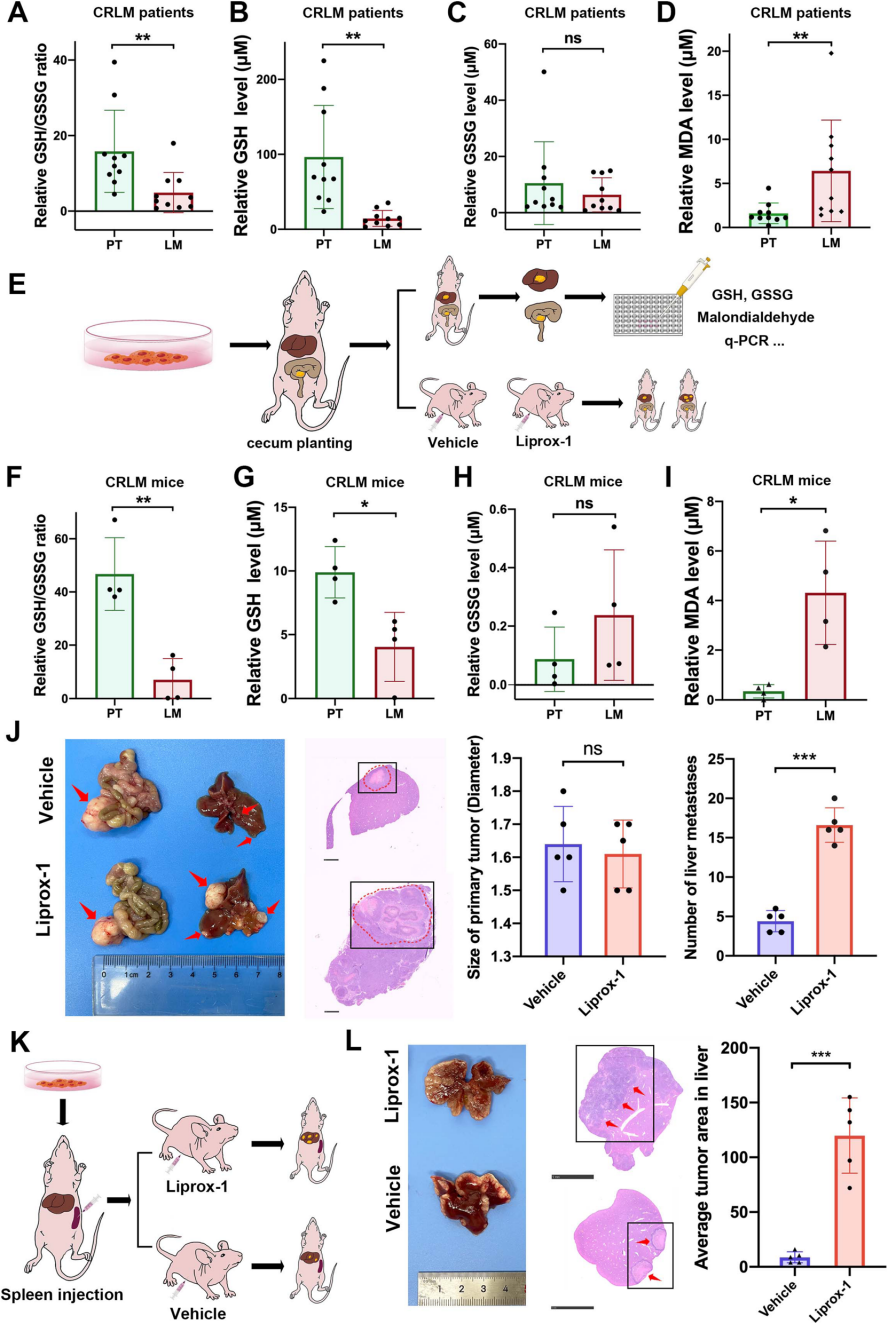
Lipid peroxidation restrains colorectal liver colonization. **A-C** Relative GSH/GSSG ratio (**A**), GSH level (**B**), and GSSG level (**C**) in paired primary tumors (PTs) and liver metastases (LMs) from CRLM patients. **D** Relative MDA levels in paired PTs and LMs from CRLM patients. **E** Schematic diagram of orthotopic CRLM mouse model establishment and subsequent sample arrangement. **F–H** Relative GSH/GSSG ratio (**F**), GSH level (**G**), and GSSG level (**H**) in paired PTs and LMs from the orthotopic CRLM mouse model. **I** Relative MDA levels in paired PTs and LMs from the orthotopic CRLM mouse model. **J** Representative bright field images, HE staining of LMs (scale bar, 1 mm), quantification of PT size and LM number derived from orthotopic CRLM mouse models treated with liproxstatin-1 or vehicle. **K** Schematic diagram of splenic injection CRC liver colonization mouse model establishment. **L** Representative bright field images, HE staining (scale bar, 5 mm), and average tumor area derived from the CRC liver colonization mouse model treated with liproxstatin-1 or vehicle. Significance was calculated by two-tailed ratio t test (**A-D**, **F-I**, right of **J** and **L**). *p* value < 0.001 (***), *p* value < 0.01 (**), *p* value < 0.05 (*), ns (not significant)

Oxidative stress caused by lipid peroxidation poses great threats to metastatic tumor cells in distant organs. To evaluate whether CRC cells experience higher oxidative stress when colonizing in the liver, we tested the GSH/GSSG ratio in paired PTs and LMs from 10 CRLM patients. LMs showed a lower GSH/GSSG ratio than PTs (*p* = 0.0077) (Fig. 1A). In particular, GSH levels were significantly lower in LMs than in PTs (*p* = 0.0028), while GSSG levels showed no significant difference (*p* = 0.3144) (Fig. 1B, C). We then tested the MDA concentration to examine the lipid peroxidation level, and LMs also exhibited higher MDA concentrations than PTs (*p* = 0.0262) (Fig. 1D).

To further verify the differences in the GSH/GSSG ratio and MDA concentration between PTs and LMs, we next established an in vivo orthotopic CRC model with spontaneous liver metastasis in BALB/c nude mice (Fig. 1E). After cecal planting, liver metastasis subsequently developed in 7 weeks, and the GSH/GSSG ratio and MDA concentration in PTs and LMs were measured. Consistent with clinical samples, a significantly lower GSH/GSSG ratio (*p* = 0.0039), lower GSH level (*p* = 0.0411), and higher MDA concentrations (*p* = 0.0425) were observed in LMs than in PTs, and no significant difference was observed in the GSSG level (*p* = 0.1433) (Fig. 1F-I), thereby recapitulating our observed profile of oxidative stress (GSH/GSSG ratio and MDA) in CRLM patient samples. Together, the above results suggested that CRC cells disseminated in the liver experienced higher levels of lipid peroxidation than those in the primary tumor.

Then we investigated the functional effect of lipid peroxidation during CRC liver metastasis in two CRLM mouse models, the abovementioned orthotopic CRLM mouse model and a CRC liver colonization mouse model by splenic injection. After CRC cell implantation, the mice were treated with vehicle or liproxstatin-1 (a lipid peroxidation inhibitor) daily by intraperitoneal injection. In orthotopic CRLM mice, liproxstatin-1 significantly increased the tumor volume of LMs (*p* < 0.0001) but had little effect on growth of PTs (*p* = 0.6733) (Fig. 1J). In the CRC liver colonization mouse model, liproxstatin-1 also significantly increased the tumor volume of LMs (*p* < 0.0001) (Fig. 1K, L). The above results suggested that inhibition of lipid peroxidation markedly promoted CRC liver colonization in different mouse models.

### AADAC is upregulated in CRC liver metastases and associated with poor prognosis

**Fig. 2:**
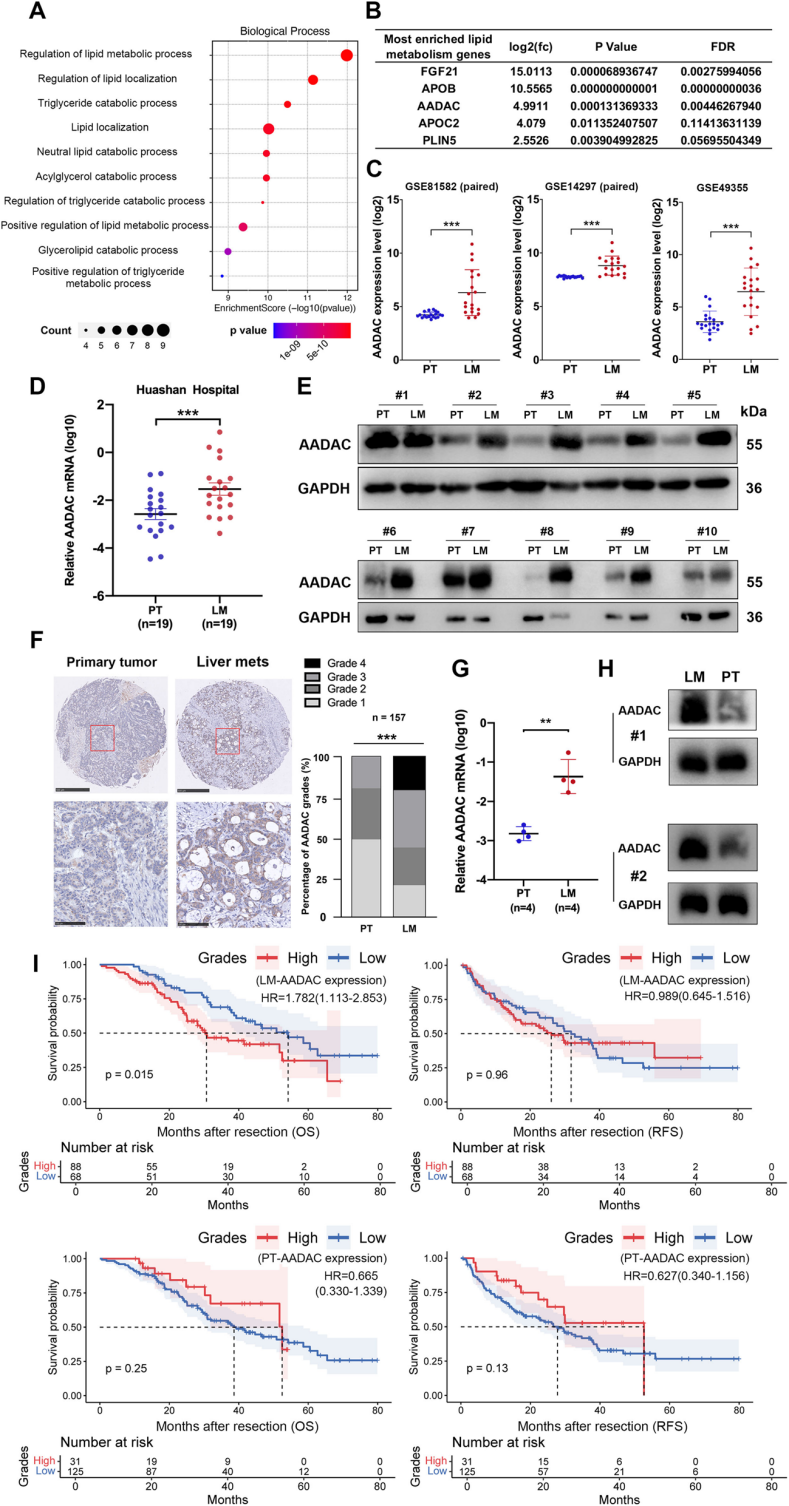
AADAC is upregulated in CRC liver metastases and associated with poor prognosis. **A** GO analysis of the 13 most enriched lipid metabolism-related genes upregulated in LMs. **B** Fold change, *p* value and FDR of the 5 most enriched genes involved in lipid metabolic processes. **C** Bioinformatic analysis comparing AADAC expression levels between paired PTs and LMs from CRLM patients in 3 GEO datasets: GSE81582 (*n* = 19), GSE14297 (*n* = 18), and GSE49355 (*n* = 20). **D** Relative AADAC mRNA levels in paired PTs and LMs from CRLM patients (*n* = 19). **E** Protein expression of AADAC in paired PTs and LMs from CRLM patients (*n* = 10). **F** IHC staining of AADAC expression in paired PTs and LMs from CRLM patients (*n* = 157). The upper scale bar represents 500 μm. The lower scale bar represents 100 μm. **G-H** Relative mRNA (**G**) and protein (**H**) levels of AADAC in paired PTs and LMs from an orthotopic CRLM mouse model (*n* = 4). **I** Kaplan–Meier plots of OS and RFS after hepatectomy in CRLM patients stratified by AADAC expression in LMs and PTs. Significance was calculated by a two-tailed ratio t test (**C-D**, right of **F**, **G**). *p* value < 0.001 (***), *p* value < 0.01 (**), *p* value < 0,05 (*), ns (not significant)

During the process of lipid peroxidation, polyunsaturated fatty acids (PUFAs) are first biosynthesized by enzymes such as acyl-CoA synthetase long chain family member 4 (ACSL4) and lysophosphatidylcholine acyltransferase 3 (LPCAT3), then they are converted into hydroperoxides by a series of lipid enzymes and cause damage to the cellular lipid bilayer [26]. This indicates that certain lipid metabolism-related genes may play a role in promoting metastatic CRC survival through elevated oxidative stress in the liver. Hence, we performed RNA-seq on paired PTs and LMs from 3 CRLM patients, and paired differential analysis between PTs and LMs revealed a set of differentially expressed genes (DEGs). Among 243 upregulated genes in LMs, we identified 13 genes involved in lipid metabolism (Supplementary Fig. 1A, B), and enrichment analysis showed that these genes played roles in several lipid metabolic processes (Fig. 2A) (Supplementary Fig. 1C, D). Among the above 13 genes, 5 were most frequently enriched in lipid metabolic processes, which contained 3 secretary factors (FGF21, APOB, and APOC2). Among the 5 genes, AADAC was a lipid enzyme overexpressed more than 4 times in LMs compared to PTs, indicating its potential role in lipid peroxidation mentioned above (Fig. 2B). Then, the expression of AADAC was compared between paired PTs and LMs from 3 independent gene expression profiling datasets (GSE81582, GSE14297, GSE49355), and the results confirmed that AADAC was significantly upregulated in LMs compared with its counterpart in PTs (Fig. 2C).

We then collected another set of paired PT and LM samples from 19 CRLM patients in our center and carried out qRT-PCR and immunoblotting to measure the expression of AADAC. Consistently, both the mRNA and protein levels of AADAC in LMs were significantly higher than those in PTs (qRT-PCR *p* = 0.0003) (Fig. 2D, E) (Supplementary Fig. 1E). AADAC expression evaluated by IHC in a CRLM TMA cohort also confirmed the significant upregulation of AADAC in LMs compared with paired PTs (*n* = 157, *p* < 0.0001) (Fig. 2F). Finally, AADAC mRNA and protein expression was also compared in PTs and LMs from an orthotopic CRLM mouse model. Consistently, LMs indeed showed higher expression of AADAC than PTs (qRT-PCR *p* < 0.0017) (Fig. 2G, H).

Survival analysis of the CRLM TMA cohort showed that CRLM patients with higher AADAC expression (higher IHC score) in LMs showed significantly shorter OS (*p* = 0.015, HR = 1.782) after hepatectomy, while its association with RFS (*p* = 0.96, HR = 0.989) was not significant. In contrast, the expression of AADAC in PTs had no impact on either OS (*p* = 0.25, HR = 0.665) or RFS (*p* = 0.13, HR = 0.627). Survival analyses in colorectal cancer (*p* = 0.911, HR = 0.98) and colon cancer (*p* = 0.794, HR = 1.05) from the TCGA cohort also showed that the expression of AADAC in primary tumors had no association with OS (Fig. 2I) (Supplementary Fig. 1F, G). In addition, although associations between AADAC expression level in LMs and number of LMs, size of LMs, and clinical risk scores (CRS) evaluated by Pearson’s χ2 tests were not significant, patients with higher AADAC expression in LMs still had a larger proportion to develop larger number (52.1%), greater size (70.6%), and higher CRS (61.3%) than those with lower AADAC expression (Supplementary Table 1). Spearman correlation analysis showed that the regional lymph node involvement stage (N classification) of PTs was positively correlated with the AADAC expression level in LMs (LM-AADAC) (*p* = 0.042) (Supplementary Table 2). Moreover, univariate Cox regression analyses showed that LM-AADAC (*p* = 0.015, HR = 1.782), N classification (*p* = 0.009, HR = 1.220), number of liver metastases (*p* = 0.037, HR = 1.668), metastases in bilateral liver lobes (*p* = 0.018, HR = 1.735), and recurrence states (*p* = 0.004, HR = 2.082) were significantly related to OS (Supplementary Table 3), while only LM-AADAC (*p* = 0.007, HR = 1.976), N classification (*p* = 0.032, HR = 1.183), and recurrence states (*p* < 0.005, HR = 2.611) were significantly related to OS in further multivariate Cox regression analyses (Supplementary Table 4). Altogether, these data suggested that AADAC was upregulated in LMs, and that AADAC overexpression in LMs was closely related to poor prognosis.

### AADAC promotes CRC proliferation and liver colonization

**Fig. 3:**
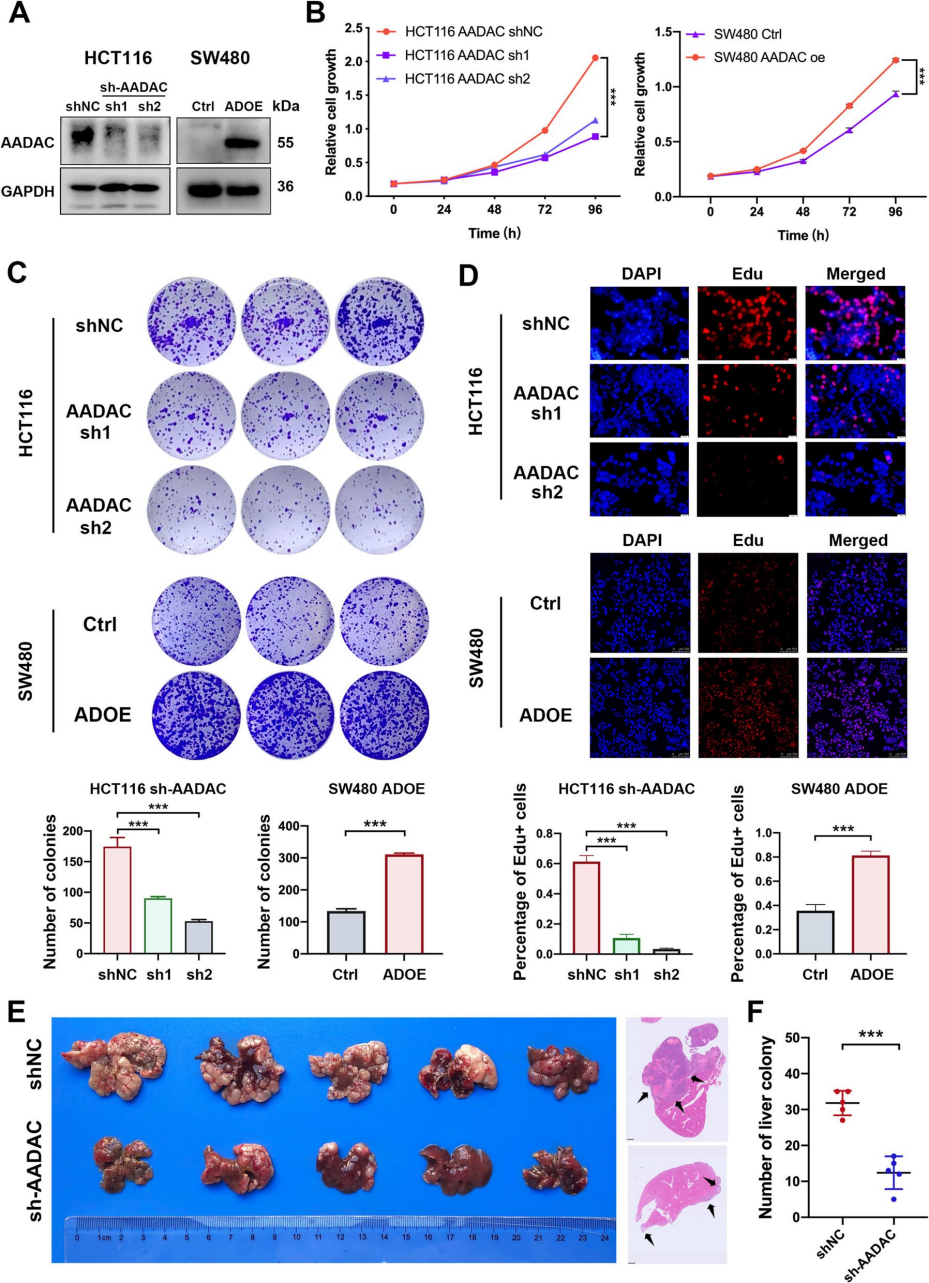
AADAC promotes CRC proliferation and liver colonization. **A** Protein expression of AADAC in sh-AADAC HCT116 cells and ADOE SW480 cells. **B** The proliferation ability of sh-AADAC and ADOE cells. **C** The colony formation ability of sh-AADAC and ADOE cells. **D** Representative fluorescent images and relative quantification of EdU staining in sh-AADAC and ADOE cells. **E** Representative images and HE staining (scale bar, 1 mm) derived from CRC liver colonization mouse model with shNC and sh-AADAC cells. **F** Quantification of liver colonies in (**E**). Data are shown as the mean ± SD. Significance was calculated by two-way ANOVA (**B**) and two-tailed ratio t test (**C-F**). *p* value < 0.001 (***), *p* value < 0.01 (**), *p* value < 0,05 (*), ns (not significant)

To investigate the oncogenic role of AADAC, we knocked down and overexpressed AADAC in the CRC cell lines HCT116 and SW480, respectively (Fig. 3A). In functional assays, deletion of AADAC in HCT116 cells dramatically impaired the proliferation (*p* < 0.001) and colony formation ability (*p* < 0.001), while ectopic overexpression of AADAC in SW480 cells pronouncedly enhanced proliferation (*p* < 0.001) and colony formation ability (*p* < 0.001) (Fig. 3B, C). Consistently, the Edu staining assay confirmed that compared to their control cells, AADAC-oe (ADOE) SW480 cells were significantly more active in DNA replication (*p* < 0.001), while sh-AADAC HCT116 cells showed decreased DNA replication activity (*p* < 0.001) (Fig. 3D). In the CRC liver colonization model, mice injected with sh-AADAC HCT116 cells exhibited a markedly decreased number of liver colonies compared to those injected with shNC cells (*p* < 0.001) (Fig. 3E, F). These results indicated that AADAC played a critical role in CRC proliferation and liver colonization.

### AADAC reduces lipid peroxidation

**Fig. 4:**
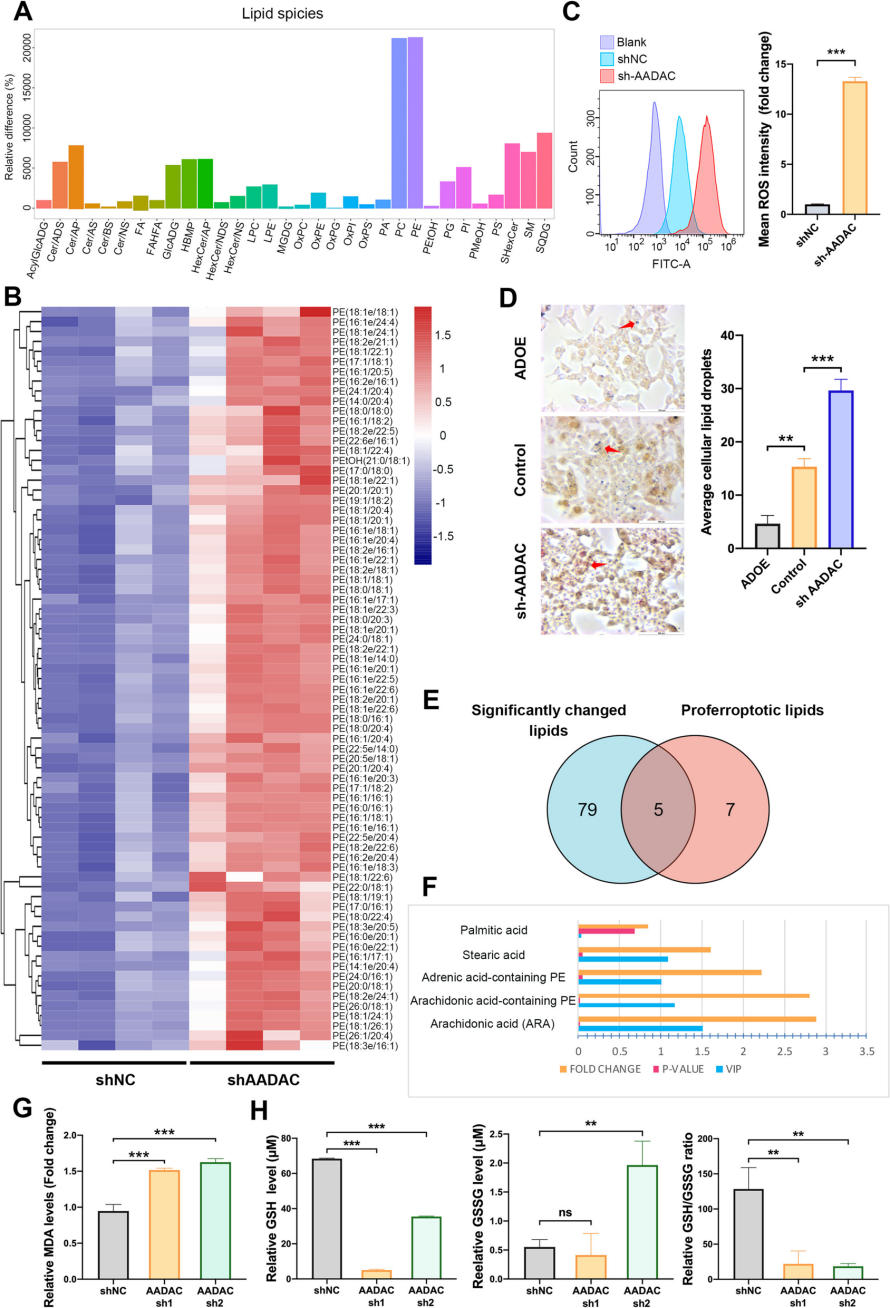
AADAC reduces lipid peroxidation. **A** Significantly enriched lipid species detected by untargeted lipidomic analysis in sh-AADAC HCT116 cells compared to shNC cells. **B** Heatmap of PUFA-containing PEs significantly enriched in sh-AADAC HCT116 cells. **C** Representative ROS levels in shNC and sh-AADAC HCT116 cells. **D** Representative images and quantification of lipid droplets in control, sh-AADAC, and AADAC-oe (ADOE) HCT116 cells (scale, 100 μm). **E** Venn diagram of significantly enriched and proferroptotic lipid species among 84 upregulated lipids in sh-AADAC cells. **F** Fold change, *p* value, and VIP value of 5 proferroptotic lipid species in (**E**). Fold change > 1.5; FDR-related *p* value < 0.05; VIP value of lipidomic analysis > 1. **G** Relative cellular MDA levels in shNC and sh-AADAC HCT116 cells treated with 15 μM erastin. **H** Relative levels of GSH, GSSG, and the GSH/GSSG ratio in sh-AADAC HCT116 cells. Data are shown as the mean ± SD. Significance was calculated by a two-tailed ratio t test (right of **C**, left of **D**, **G-H**). *p* value < 0.001 (***), *p* value < 0.01 (**), *p* value < 0,05 (*), ns (not significant)

To determine whether AADAC is involved in regulating lipid peroxidation, we conducted untargeted lipidomic analysis of shNC and sh-AADAC HCT116 cells. Among the 32 most upregulated lipid species, phosphatidylethanolamines (PEs) emerged as one of the most enriched lipid species. Further profiling of these upregulated PEs showed that PUFA-containing lipids including oxygenated PEs, which play indispensable roles in lipid peroxidation as mentioned before, were significantly upregulated in sh-AADAC HCT116 cells (Fig. 4A, B). Consistently, a significant increase in lipid reactive oxygen species (ROS) was observed in sh-AADAC cells compared to shNC cells, confirming the role of AADAC in reducing lipid peroxidation (Fig. 4C). It has been reported that lipid droplets (LDs) act as a form of storage for excess PUFAs in lipid peroxidation [27]. Compared to control cells, we observed significantly increased cellular LDs in sh-AADAC HCT116 cells and decreased LDs in ADOE cells (Fig. 4D). This indicated that AADAC not only reduced the accumulation of PUFA-containing PEs, but also restrained the formation of LDs.

Notably, dysfunctional ROS scavenging and lipid peroxidation play indispensable roles in ferroptosis. Among the 84 most enriched lipid species, including PEs and other fatty acids (FAs), we noted that 5 species were reported to promote ferroptosis [27, 28], and only 4 out of the 5 species remained statistically enriched after further assessment (variable importance in the projection (VIP) value of lipidomic analyses > 1; *p* value < 0.05; fold change > 1.5) (Fig. 4E, F). Furthermore, we observed higher MDA levels in sh-AADAC HCT116 cells (*p* < 0.001) (Fig. 4G) and reduced MDA levels in ADOE SW480 cells (*p* < 0.01) (Supplementary Fig. 2A). Consistently, deletion of AADAC resulted in a significant decrease in GSH levels (*p* < 0.001) and the GSH/GSSG ratio (*p* < 0.01) (Fig. 4H). As expected, overexpression of AADAC reversed the above effects on GSH levels (*p* < 0.001) and the GSH/GSSG ratio (*p* < 0.001), further confirming the anti-lipid peroxidation role of AADAC (Supplementary Fig. 2B).

### AADAC upregulates SLC7A11 to suppress ferroptosis

**Fig. 5:**
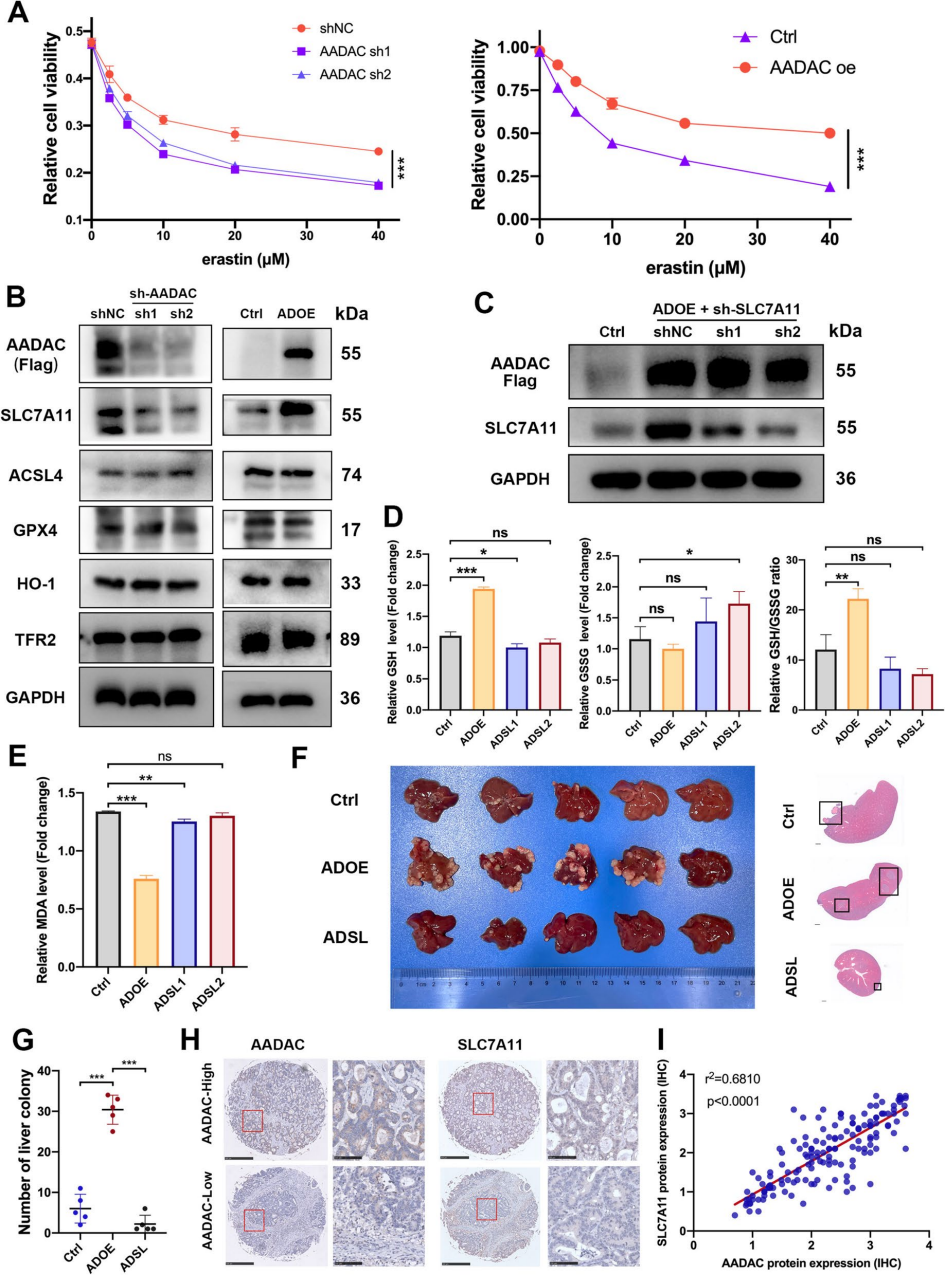
AADAC upregulates SLC7A11 to suppress ferroptosis. **A** Cell viability of sh-AADAC HCT116 and ADOE SW480 cells treated with graded concentrations of erastin. **B** Protein expression of ferroptosis-related genes in sh-AADAC HCT116 cells and ADOE SW480 cells. **C** Protein expression of SLC7A11 in control, ADOE, and ADOE + sh-SLC7A11 (ADSL) SW480 cells. **D** Relative levels of GSH, GSSG, and the GSH/GSSG ratio in control, ADOE, and ADSL SW480 cells. **E** Relative MDA levels in control, ADOE, and ADSL SW480 cells treated with 15 μM erastin. **F-G** Representative images, HE staining (scale bar, 1 mm) (**F**) and quantification (**G**) of liver colonies from CRC liver colonization mice model with different SW480 cells (control, ADOE, and ADSL). **H** Representative IHC staining of AADAC and SLC7A11 expression in the LMs of CRLM patients (left scale bar, 500 μm; right scale bar, 100 μm). **I** Pearson correlation between IHC H scores of AADAC and SLC7A11 expression in LMs. Data are shown as the mean ± SD. Significance was calculated by two-way ANOVA and two-tailed ratio t test (**A**, **D**, **E**, **G**). *p* value < 0.001 (***), *p* value < 0.01 (**), *p* value < 0,05 (*), ns (not significant)

To investigate the effect of AADAC on the sensitivity of cells to ferroptosis, we administered graded concentrations of erastin, an agonist that specifically induces ferroptosis, to sh-AADAC and shNC HCT116 cells. Compared to shNC HCT116 cells, remarkably attenuated cell viability was observed in sh-AADAC cells. In contrast, overexpression of AADAC in SW480 cells partially abrogated the ferroptotic cell death at different concentrations of erastin. (Fig. 5A).

The sensitivity of cells to ferroptosis is determined by various factors, namely, GSH biosynthesis, iron metabolism, PUFA metabolism, and so on. To unveil the mechanism by which AADAC protects CRC liver metastasis against ferroptosis, we next evaluated the expression of ferroptosis-related genes in sh-AADAC and ADOE CRC cell lines. Among these genes, SLC7A11, a vital ferroptosis regulator that is involved in cysteine-glutamate metabolism, exhibited a decrease in sh-AADAC, and an upregulation in ADOE cell lines. No significant changes were observed in other genes, such as ACSL4, GPX4, HO-1 and TFR2 (Fig. 5B). To verify whether SLC7A11 mediated the anti-ferroptosis effect of AADAC, we first constructed ADOE + sh-SLC7A11 (ADSL) SW480 cells (Fig. 5C). Overexpression of AADAC ameliorated oxidative stress as it caused a significant increase in the GSH/GSSG ratio (*p* < 0.001) and reduced MDA levels (*p* < 0.001), and further depletion of SLC7A11 abrogated this effect (Fig. 5E).

To further determine whether SLC7A11 mediated the promoting effect of AADAC on CRC liver colonization, a CRC liver colonization model was used to compare the colonization formation ability between Ctrl (AADAC-control), ADOE and ADSL SW480 cells. The results showed that mice injected with ADOE SW480 cells developed more liver colonies than those injected with Ctrl cells (*p* < 0.001), and depletion of SLC7A11 in ADOE cells potently reduced metastatic colonies (*p* < 0.001) (Fig. 5F, G). In IHC analysis of clinical CRLM samples, the expression of AADAC was positively correlated with that of SLC7A11 (*p* < 0.0001) (Fig. 5H, I). Collectively, this evidence revealed that AADAC upregulated SLC7A11 to suppress ferroptosis, thus promoting CRC liver colonization.

### AADAC upregulates SLC7A11 by activating NRF2

**Fig. 6:**
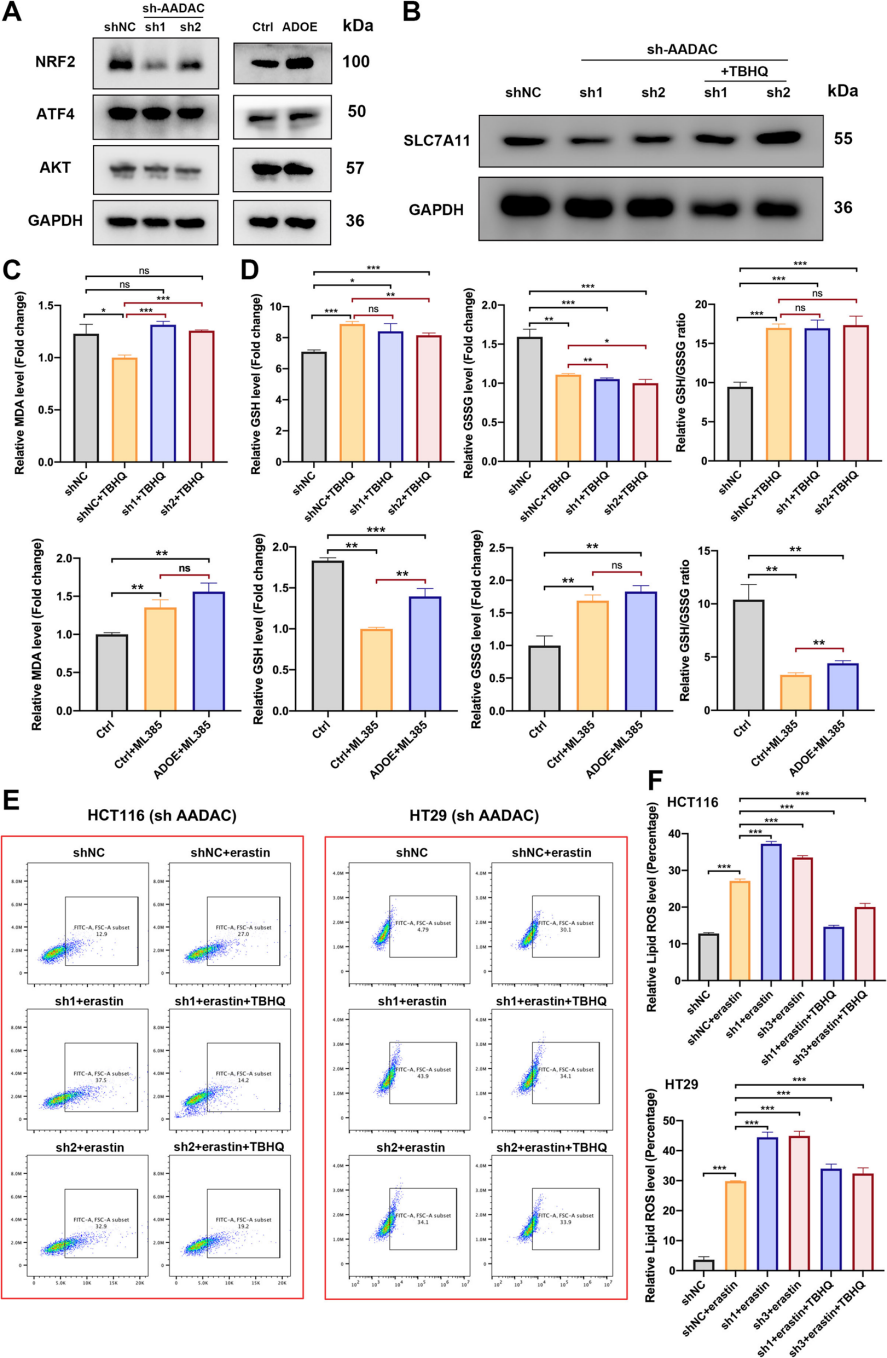
AADAC upregulates SLC7A11 by activating NRF2. **A** Protein expression of SLC7A11 upstream genes in sh-AADAC cells. **B** Protein expression of SLC7A11 in shNC, sh-AADAC and sh-AADAC + 10 μM TBHQ HCT116 cells. **C** Relative MDA levels in shNC, sh-AADAC, and sh-AADAC + 10 μM TBHQ HCT116 cells treated with 15 μM erastin, and in SW480 cells (control, ADOE, ADOE + ML385) treated with 15 μM erastin. **D** Relative GSH level, GSSG level and GSH/GSSG ratio of HCT116 cells (shNC, shNC + 10 μM TBHQ, and sh-AADAC + 10 μM TBHQ) and SW480 cells (control, control + ML385, ADOE + ML385). **E** Relative levels of lipid ROS in HCT116 and HT29 cells pretreated with or without 10 μM TBHQ for 1 h, and with 15 μM erastin for 48 h. **F** Data are shown as the mean ± SD. Significance was calculated by a two-tailed ratio t test (**C**, **D**, **F**). *p* value < 0.001 (***), *p* value < 0.01 (**), *p* value < 0,05 (*), ns (not significant)

**Fig. 7:**
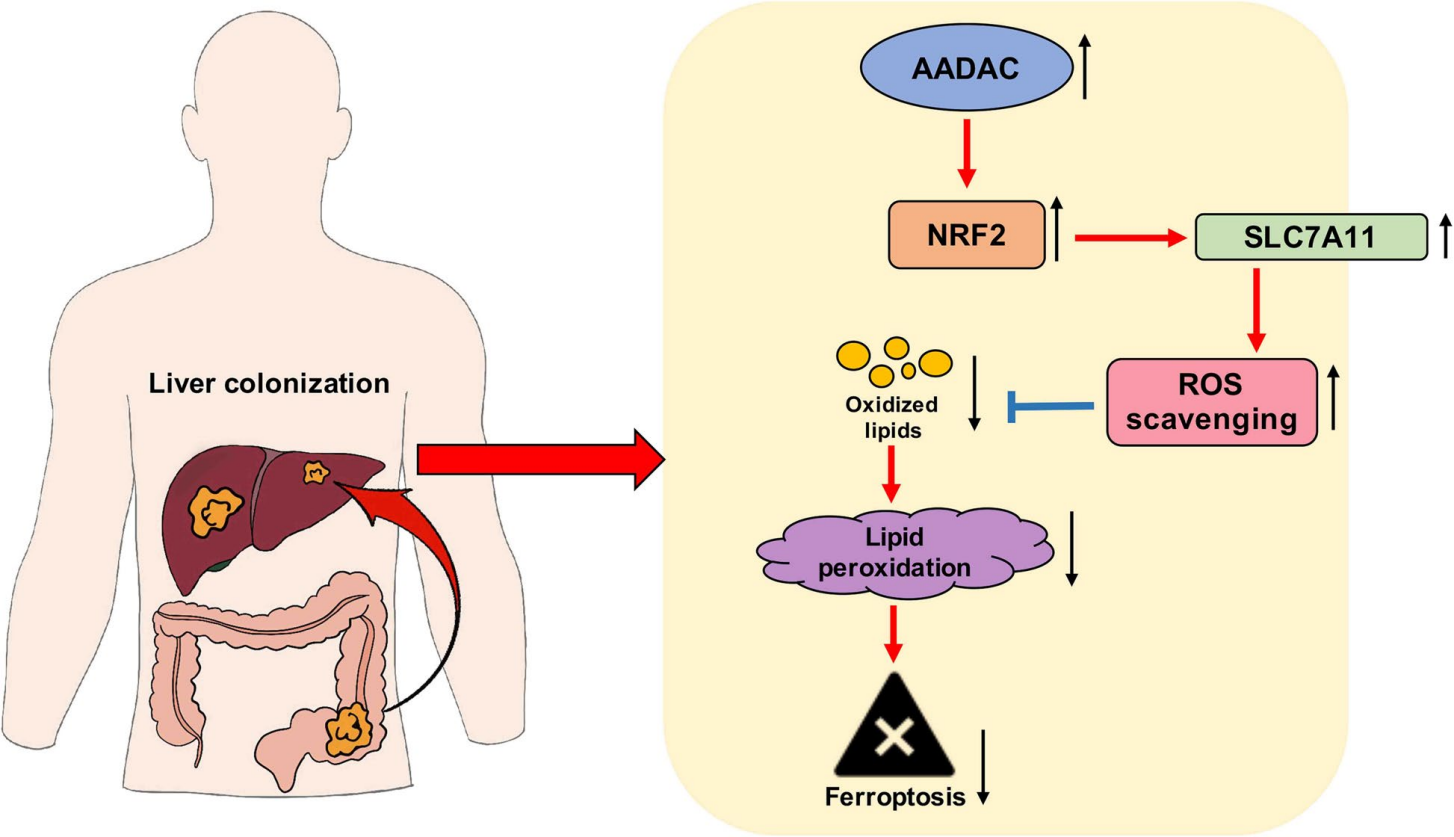
Schematic diagram of AADAC inhibition of lipid peroxidation and ferroptosis via NRF2/SLC7A11 axis

To further understand the underlying mechanism by which AADAC upregulated SLC7A11, we analyzed canonical SLC7A11 upstream regulators. Immunoblotting showed that the expression of NRF2 was markedly downregulated after deletion of AADAC, and in contrast, overexpression of AADAC led to a significant increase in NRF2 expression. The expression of other potential regulators, such as ATF4 and AKT, did not show remarkable changes (Fig. 6A, B). Then we treated sh-AADAC cells with TBHQ, a metabolite that induces NRF2 activation, and found that TBHQ treatment significantly rescued SLC7A11 expression in sh-AADAC cells (Fig. 6B). These results indicated that NRF2 mediated the regulatory role of AADAC on SLC7A11 expression (Fig. 7).

To verify whether NRF2 mediated the suppressive role of AADAC in lipid peroxidation and ferroptosis, we treated sh-AADAC cells with TBHQ and ML385, an NRF2 inhibitor. TBHQ treatment significantly reduced MDA levels and increased the GSH/GSSG ratio of sh-AADAC cells (Fig. 6C, D), while ML385 treatment significantly increased MDA level and reduced GSH/GSSG ratio in ADOE cells (Fig. 6C, D). Consistently, depletion of AADAC in HCT116 and HT29 cells caused significant increase in cellular lipid ROS, indicating failure to protect cells from erastin-induced cell death, while administration of TBHQ efficiently rescued this effect (Fig. 6E-F). Taken together, these results demonstrated that AADAC protected CRC liver colonization from ferroptosis through the NRF2-SLC7A11 axis (Fig. 7).

## Discussion

A few recent studies have revealed that oxidative stress restrains the metastasis of melanoma [19, 21]. In this study, we found that compared with primary tumors, metastatic CRC cells experienced increased oxidative stress, which attenuated their colonization in the liver. We also identified upregulated AADAC in liver metastases as a protector against lipid peroxidation to promote CRC liver colonization, and uncovered a hitherto unrecognized regulatory coupling between AADAC and ferroptosis. Mechanistically, AADAC upregulated SLC7A11 via activation of NRF2 to reduce lipid peroxidation, hence protecting liver-colonized CRC from ferroptosis. In addition, high expression of AADAC was closely associated with poor postoperative survival of CRLM patients. These results revealed that AADAC functions as a novel ferroptosis suppressor and provided a potential approach against CRC liver colonization by targeting AADAC.

An understanding of cancer metastasis has been established, as primary tumors have a propensity to reprogram their metabolic processes or to be reshaped by the organ milieu where they colonize, hence transferring into metastatic organ-like metabolic patterns [29, 30]. However, whether different types of cancer have the same reprogramming pattern when colonizing in the same organ, or how different subpopulations of one type of primary cancer are reshaped, remain largely unknown. Previous studies addressing metabolic alterations in CRC liver metastasis have shown that aldolase B-mediated fructose metabolic reprogramming and cholesterol metabolic changes play vital roles in CRC liver metastasis and colonization. Fatty acid metabolism, triglyceride metabolism and other metabolic processes were also been identified to be active in liver metastasis [22, 29]. Our findings about differentially expressed genes in CRC liver metastasis and their relevant metabolic processes partially overlapped with previous reports, but did not equate, indicating that different subpopulations of one type of primary cancer might favor different milieus of a metastatic organ or have variant plasticity, which awaits further investigation.

As the most important finding of our study, AADAC, a lipid enzyme normally active in the liver, along with its metabolic process, was found to be more enriched in liver metastases than in their paired primary tumors. Existing studies concerning AADAC have uncovered its variant roles in different diseases. In type 2 diabetes, elevated expression of AADAC in vascular smooth muscle cells altered lipid metabolism and protected patients from cardiovascular diseases [31]. In tuberculosis treated with rifapentine, patients carrying variants of the AADAC gene have a lower drug clearance rate [32]. A previous study also revealed that overexpression of AADAC in hepatocytes increased triacylglycerol mobilization and partly delivered it to beta-oxidation [33]. However, whether AADAC plays a role in cancer progression, especially cancer metastasis, has not been well investigated. In addition to regulation of previously described lipid metabolic and triglyceride catabolic processes, our study unveiled the oncogenic, especially pro-colonization function of AADAC in CRLM for the first time. Moreover, we showed a newly identified role of AADAC in regulating lipid metabolism, as it reduced ROS accumulation to inhibit lipid peroxidation and ferroptosis. Further investigation revealed that AADAC reduced lipid peroxidation via SLC7A11-dependent scavenging of ROS, thus protecting metastatic CRC cells from ferroptosis. Our work helps extend the preceding knowledge of AADAC and demonstrates its function in CRLM.

Previous studies have reported the antitumor role of ferroptosis. For metastatic melanomas, lymph protects their metastasis when undergoing ferroptosis [19]. For lung cancer, RNA-binding protein RBMS1-mediated ferroptosis evasion promoted its development [34]. Here we uncovered the inhibitory roles of lipid peroxidation and ferroptosis in CRC liver colonization, yet what we discovered was not mechanistically equivalent to previous findings, indicating that how cancer cells escape from ferroptosis is context dependent.

To summarize, our study showed that the CRC liver metastases display higher level of oxidative stress derived from lipid peroxidation than primary tumors. AADAC functions as a novel ferroptosis suppressor via NRF2/SLC7A11 axis-mediated inhibition of lipid peroxidation, to promote CRC liver colonization. Taken together, the AADAC/NRF2/SLC7A11 axis is a promising target for the future development of CRLM therapies.

## Conclusions

In summary, this study uncovered the poor clinical prognosis and anti-ferroptosis role of AADAC in colorectal cancer liver colonization for the first time. Our findings not only suggest a novel indicator for identifying CRLM patients who may have poor outcomes, but also provide potential therapeutic targets by unveiling the underlying mechanisms through which AADAC inhibits ferroptosis and promotes colorectal liver colonization. Together, this research expands the options available to maximize the treatment efficacy of CRLM therapy.

## Supplementary Information

**Additional file 1.**

## Data Availability

All data supporting the findings of this study are available from the corresponding author on reasonable request.

## Abbreviations

CRLM: Colorectal liver metastasis
CRC: Colorectal cancer
GEO: Gene expression omnibus
TCGA: The Cancer Genome Atlas
PUFAs: Polyunsaturated fatty acids
AADAC: Arylacetamide deacetylase
SLC7A11: Solute carrier family 7 member 11
NRF2: Nuclear factor erythroid-2
ACSL4: Acyl-CoA synthetase long chain family member 4
LPCAT3: Lysophosphatidylcholine acyltransferase 3
PE: Phosphatidylethanolamine
ADOE: AADAC overexpression
ADSL: ADOE + sh SLC7A11
