# Correction: AADAC protects colorectal cancer liver colonization from ferroptosis through SLC7A11‑dependent inhibition of lipid peroxidation

**DOI:** 10.1186/s13046-022-02508-w

**Published:** 2022-10-25

**Authors:** Rongquan Sun, Zhifei Lin, Xiangyu Wang, Lu Liu, Meisi Huo, Rui Zhang, Jing Lin, Chao Xiao, Yitong Li, Wenwei Zhu, Lu Lu, Jubo Zhang, Jinhong Chen

**Affiliations:** 1 Department of General Surgery, Cancer Metastasis Institute, Huashan Hospital, Fudan University, No.12, Middle Urumqi Road, 200040 Shanghai, China; 2 Department of Infectious Diseases, Huashan Hospital, Fudan University, 200040 Shanghai, China

**Correction**: ***J Exp Clin Cancer Res*** **41, 284 (2022) https://doi.org/10.1186/s13046-022-02493-0**

Following publication of the original article [1], an error was identified in Fig. 6; specifically:

- Figure 6E: The flow cytometry image of group HT29 sh2 + erastin
- Figure 6F: The group label “sh3” should be corrected into “sh2”

**Fig. 6:**
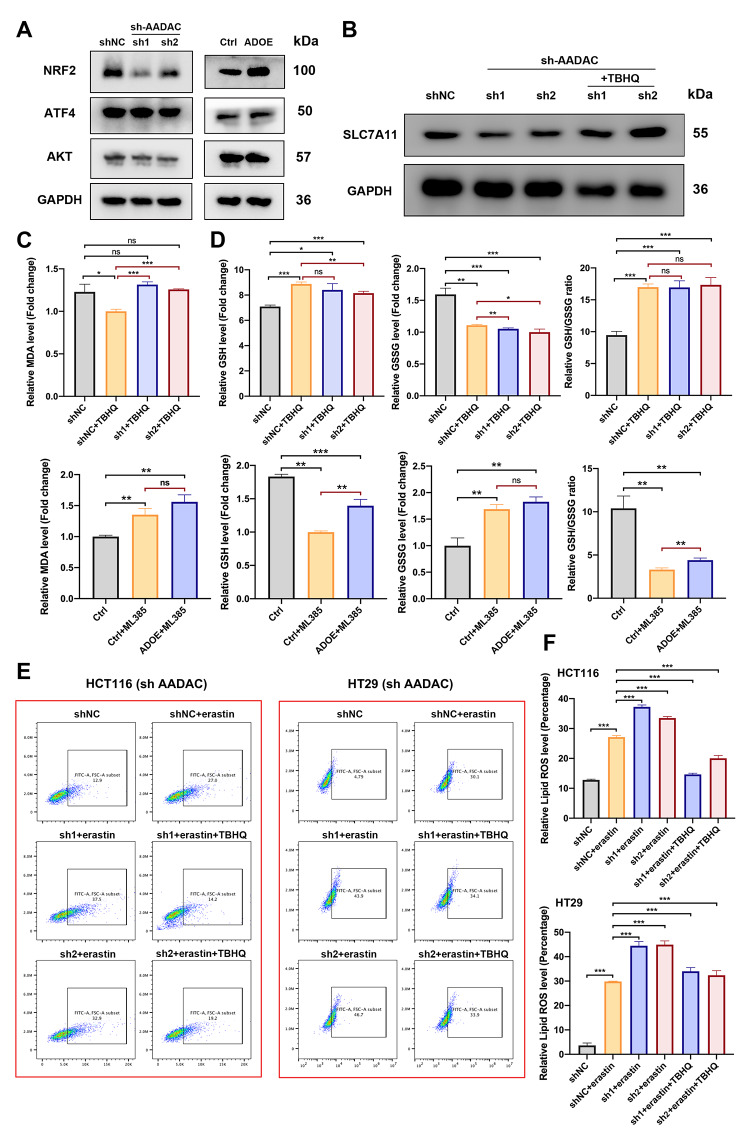
AADAC upregulates SLC7A11 by activating NRF2. **A** Protein expression of SLC7A11 upstream genes in sh-AADAC cells. **B** Protein expression of SLC7A11 in shNC, sh-AADAC and sh-AADAC + 10 µM TBHQ HCT116 cells. **C** Relative MDA levels in shNC, sh-AADAC, and sh-AADAC + 10 µM TBHQ HCT116 cells treated with 15 µM erastin, and in SW480 cells (control, ADOE, ADOE + ML385) treated with 15 µM erastin. **D** Relative GSH level, GSSG level and GSH/GSSG ratio of HCT116 cells (shNC, shNC + 10 µM TBHQ, and sh-AADAC + 10 µM TBHQ) and SW480 cells (control, control + ML385, ADOE + ML385). **E** Relative levels of lipid ROS in HCT116 and HT29 cells pretreated with or without 10 µM TBHQ for 1 h, and with 15 µM erastin for 48 h. **F** Data are shown as the mean ± SD. Significance was calculated by a two-tailed ratio t test (**C, D, F**). *p* value < 0.001 (***), *p* value < 0.01 (**), *p* value < 0,05 (*), ns (not significant)

The correct figure is now been provided. The correction does not have any effect on the results or conclusions of the paper. The original article has been corrected.
