# Supplementary material for: AADAC protects colorectal cancer liver colonization from ferroptosis through SLC7A11-dependent inhibition of lipid peroxidation
Source: J Exp Clin Cancer Res. 2022 Sep 26;41:284. doi: 10.1186/s13046-022-02493-0 (PMC9511737; doi:10.1186/s13046-022-02493-0)
Supplement: Supplementary file 1 — Additional file 1. [file 13046_2022_2493_MOESM1_ESM.docx]

**AADAC protects colorectal cancer liver colonization from ferroptosis through SLC7A11-dependent inhibition of lipid peroxidation-supplemental materials**

Rongquan SUN^1†^, Zhifei LIN^1†^, Xiangyu WANG^1†^, Lu LIU^2^, Meisi HUO^2^, Rui ZHANG^1^, Jing LIN^1^, Chao XIAO^1^, Yitong LI^1^, Wenwei ZHU^1^, Lu LU^1^, Jubo ZHANG^2^, Jinhong CHEN^1^*

**Table of contents in supplemental materials**

| **Category** | **Label** | **content** |
| --- | --- | --- |
| **Figure** | Supplementary fig. 1 | Supplementary figure of Fig.2 |
|  | Supplementary fig. 2 | Supplementary figure of Fig.4 |
| **Table** | Supplementary Table1 | Association between AADAC expression in liver metastasis and the clinical characteristics of CRLM patients (n=157) by Pearson’s χ2 tests |
|  | Supplementary Table2 | Spearman correlation analysis of the association between AADAC expression in liver metastasis and various clinical characteristics |
|  | Supplementary Table3 | Univariate Cox regression analysis of the association between clinicopathological characteristics and post-operative survival in CRLM patients |
|  | Supplementary Table4 | Multivariate Cox regression analysis of the association between clinicopathological characteristics and post-operative survival in CRLM patients |
|  | Supplementary Table5 | Primer sequences used in research |
|  | Supplementary Table6 | Antibodies used in research |

**Methods**

Enriched analysis, immunoblotting, survival analysis, GSH/GSSG ratio detection, MDA detection were described in text contents of the paper. Pearson’s χ2 tests, Spearman correlation analysis, Univariate Cox regression analysis, multivariate Cox regression analysis by SPSS Statistics 20 were used for clinical data analyses.

**
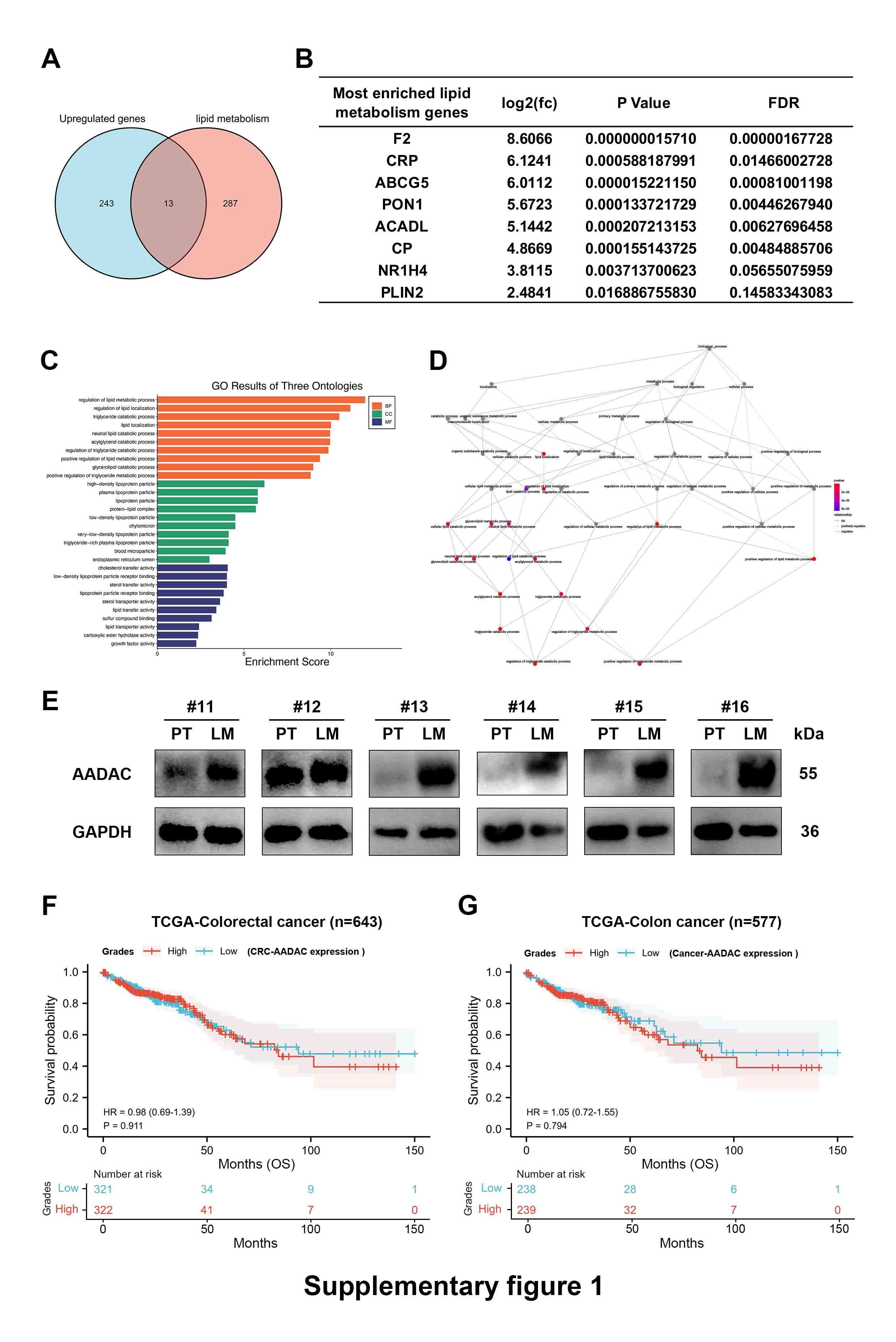
**

**Supplementary fig. 1.** (A) Venn diagram of significantly enriched lipid metabolism-related genes in 243 upregulated genes in LMs compared to PTs. (B) The other 8 genes of 13 lipid metabolism-related genes not shown in Fig2. (C) GO analysis of 13 enriched genes in (A). (D) GO plot of relationships between biological processes mediated by 13 genes in (A). (E) Protein expression of AADAC in paired PTs and LMs from CRLM patients (No.11-No.16). (F-G) Kaplan-Meier analysis of OS of patients with colorectal cancer (F) and colon cancer (G) with low and high AADAC expression from TGGA database. Log-rank test was used to determined p value. p value < 0.001 (***), p value < 0.01 (**), p value < 0,05 (*), ns (not significant).

**
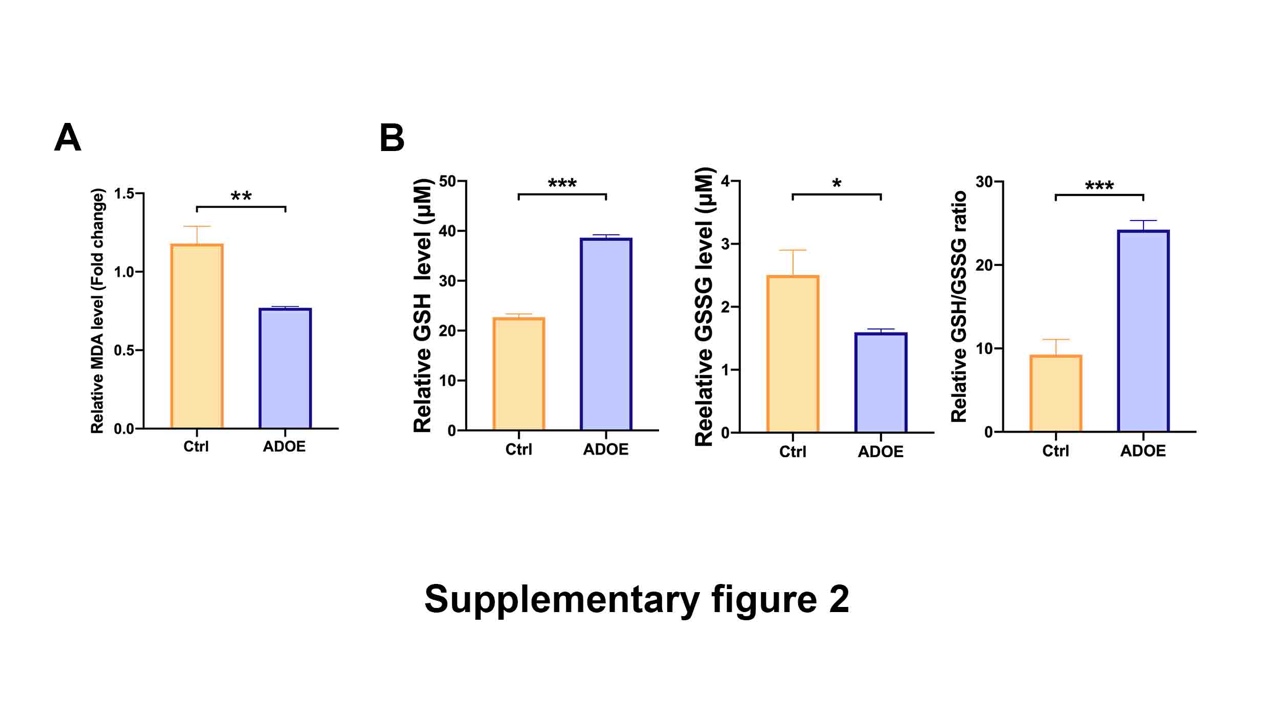
**

**Supplementary fig. 2.** (A) Relative MDA level in control and ADOE SW480 cells treated with 15 μM erastin. (B) Relative levels of GSH, GSSG and GSH/GSSG ratio of Ctrl and ADOE SW480 cells. Significance was calculated by two-tailed ratio t test (A, B). p value < 0.001 (***), p value < 0.01 (**), p value < 0,05 (*), ns (not significant).

**Supplementary Table1. Association between AADAC expression in liver metastasis and the clinical characteristics of CRLM patients (n=157) by Pearson’s χ2 tests**

| **Feature** | **Total** | **AADAC-Low (%)** | **AADAC-High (%)** | **p value** |
| --- | --- | --- | --- | --- |
| **Total** | 157 | 68 (43.3%) | 89 (56.7%) |  |
| **Age (y)** |  |  |  |  |
| < 60 | 78 (49.7%) | 32 (41%) | 46 (59%) | 0.566 |
| ≥ 60 | 79 (50.3%) | 36 (45.6%) | 43 (54.4%) |  |
| **Gender** |  |  |  |  |
| Male | 104 (66.2%) | 48 (46.2%) | 56 (53.8%) | 0.314 |
| Female | 53 (33.8%) | 20 (37.7%) | 33 (62.3%) |  |
| **number of liver metastases** |  |  |  |  |
| 1 | 64 (40.8%) | 30 (46.9%) | 34 (53.1%) | 0.455 |
| >1 | 73 (59.2%) | 38 (47.9%) | 55 (52.1%) |  |
| **Metastasis in bilateral liver lobes** |  |  |  |  |
| No | 90 (57.3%) | 43 (47.8%) | 47 (52.2%) | 0.191 |
| Yes | 67 (42.7%) | 25 (37.3%) | 42 (62.7%) |  |
| **Size of metastasis** |  |  |  |  |
| ≤ 5cm | 140 (89.2%) | 63 (45%) | 77 (55%) | 0.221 |
| > 5cm | 17 (10.8%) | 5 (29.4%) | 12 (70.6%) |  |
| **T classification** |  |  |  |  |
| Tx | 1 (0.6%) | 0 (0) | 1 (100%) | 0.745 |
| T2 | 16 (10.2%) | 7 (43.8%) | 9 (56.2%) |  |
| T3 | 36 (22.9%) | 15 (41.7%) | 21 (58.3%) |  |
| T4 | 1 (0.6%) | 1 (100%) | 0 (0) |  |
| T4a | 86 (54.8%) | 39 (45.3%) | 47 (54.7%) |  |
| T4b | 17 (10.8%) | 6 (35.3%) | 11 (64.7%) |  |
| **N classification** |  |  |  |  |
| N0 | 33 (21%) | 17 (51.5%) | 16 (48 .5%) | 0.389 |
| N1a | 15 (9.6%) | 9 (60%) | 6 (40%) |  |
| N1b | 26 (16.6%) | 12 (46.2%) | 14 (53.8%) |  |
| N1c | 36 (22.9%) | 15 (41.7%) | 21 (58.3%) |  |
| N2a | 34 (21.7%) | 11 (32.4%) | 23 (67.6%) |  |
| N2b | 13 (8.3%) | 4 (30.8%) | 9 (69.2%) |  |
| **Recurrence states** |  |  |  |  |
| No | 71 (45.2%) | 27 (38%) | 44 (62%) | 0.225 |
| Yes | 86 (54.8%) | 41 (47.7%) | 45 (52.3%) |  |
| **CRS score** |  |  |  |  |
| Low | 82 (52.2%) | 39 (47.6%) | 43 (52.4%) | 0.261 |
| High | 75 (47.8%) | 29 (38.7%) | 46(61.3%) |  |

**Supplementary Table 2. Spearman correlation analysis of the association between AADAC expression in liver metastasis and various clinical characteristics**

| **Variables** | **AADAC expression level** | |
| --- | --- | --- |
|  | **Spearman correlation** | **p value** |
| **Age** | -0.024 | 0.770 |
| **Gender** | 0.094 | 0.242 |
| **T classification** | -0.043 | 0.596 |
| **N classification** | 0.163 | 0.042 |
| **number of liver metastases** | 0.107 | 0.181 |
| **Size of metastasis** | 0.098 | 0.223 |
| **Recurrence states** | -0.109 | 0.176 |
| **CRS score** | 0.088 | 0.275 |

**Supplementary Table 3. Univariate Cox regression analysis of the association between clinicopathological characteristics and post-operative survival in CRLM patients**

| **Feature** | **p value** | **Hazard ratio (95% CI)** | **95% confidence interval** |
| --- | --- | --- | --- |
| **LM-AADAC** | 0.015 | 1.782 | 1.113~2.853 |
| **Age** | 0.130 | 0.703 | 0.445~1.110 |
| **Gender** | 0.906 | 0.972 | 0.601~1.571 |
| **T classification** | 0.095 | 1.180 | 0.972~1.432 |
| **N classification** | 0.009 | 1.220 | 1.051~1.417 |
| **number of liver metastases** | 0.037 | 1.668 | 1.031~2.700 |
| **Metastasis in bilateral liver lobes** | 0.018 | 1.735 | 1.099~2.741 |
| **Size of metastasis** | 0.556 | 1.222 | 0.627~2.382 |
| **Recurrence states** | 0.004 | 2.082 | 1.265~3.426 |
| **CRS score group** | 0.12 | 1.437 | 0.910~2.268 |
| **CRS score** | 0.183 | 1.173 | 0.928~1.482 |

**Supplementary Table 4. Multivariate Cox regression analysis of the association between clinicopathological characteristics and post-operative survival in CRLM patients**

| **Feature** | **p value** | **Hazard ratio (95% CI)** | **95% confidence interval** |
| --- | --- | --- | --- |
| **LM-AADAC** | 0.007 | 1.976 | 1.200~3.256 |
| **N classification** | 0.032 | 1.183 | 1.015~1.380 |
| **number of liver metastases** | 0.577 | 1.205 | 0.626~2.320 |
| **Metastasis in bilateral liver lobes** | 0.237 | 1.457 | 0.781~2.717 |
| **Recurrence states** | <0.005 | 2.611 | 1.560~4.370 |

**Supplementary Table 5. Primer sequences used in research**

| **Gene** | **Primer sequence** |
| --- | --- |
|  | |
| **AADAC-Forward Primer** | ATCGCTGTACCTTCTGATTGTG |
| **AADAC-Reverse Primer** | ATCGCTGTACCTTCTGATTGTG |
|  | |
| **GAPDH-Forward Primer** | GGAGCGAGATCCCTCCAAAAT |
| **GAPDH-Reverse Primer** | GGCTGTTGTCATACTTCTCATGG |
|  | |
| **AADAC sh1-Forward Primer** | CCGGCGTATCAACCAACTACAGATTCTCGAGAATCTGTAGTTGGTTGATACGTTTTTG |
| **AADAC sh1-Reverse Primer** | AATTCAAAAACGTATCAACCAACTACAGATTCTCGAGAATCTGTAGTTGGTTGATACG |
|  | |
| **AADAC sh2-Forward Primer** | CCGGCGTCCGCATATACATACCCAACTCGAGTTGGGTATGTATATGCGGACGTTTTTG |
| **AADAC sh2-Reverse Primer** | AATTCAAAAACGTCCGCATATACATACCCAACTCGAGTTGGGTATGTATATGCGGACG |
|  | |
| **SLC7A11 sh1-Forward Primer** | CCGGCCTGTCACTATTTGGAGCTTTCTCGAGAAAGCTCCAAATAGTGACAGGTTTTTG |
| **SLC7A11 sh1-Reverse Primer** | AATTCAAAAACCTGTCACTATTTGGAGCTTTCTCGAGAAAGCTCCAAATAGTGACAGG |
|  | |
| **SLC7A11 sh2-Forward Primer** | CCGGCCTGCGTATTATCTCTTTATTCTCGAGAATAAAGAGATAATACGCAGGTTTTTG |
| **SLC7A11 sh2-Reverse Primer** | AATTCAAAAACCTGCGTATTATCTCTTTATTCTCGAGAATAAAGAGATAATACGCAGG |

**Supplementary Table 6. Antibodies used in research**

| Name | Manufacturer | Catalog number |
| --- | --- | --- |
| AADAC | Proteintech | 26634-1-AP |
| NRF2 | Proteintech | 66504-1-Ig |
| ACSL4 | Proteintech | 66617-1-Ig |
| GAPDH | Proteintech | 60004-1-Ig |
| HO-1 | Proteintech | 66743-1-Ig |
| TFR2 | Abcam | Ab185550 |
| GPX-4 | Abcam | Ab125066 |
| ATF4 | Abcam | Ab184909 |
| SLC7A11 | Abcam | Ab-37185 |
| AKT | Abcam | Ab8805 |
| anti-DDDDK-Tag pAb | Abclone | AE004 |
